# Shape Matters: Impact of Mesoporous Silica Nanoparticle Morphology on Anti-Tumor Efficacy

**DOI:** 10.3390/pharmaceutics16050632

**Published:** 2024-05-08

**Authors:** Weixiang Fang, Kailing Yu, Songhan Zhang, Lai Jiang, Hongyue Zheng, Qiaoling Huang, Fanzhu Li

**Affiliations:** 1 School of Pharmaceutical Sciences, Zhejiang Chinese Medical University, Hangzhou 310053, China; 2 Libraries of Zhejiang Chinese Medical University, Zhejiang Chinese Medical University, Hangzhou 310053, China; 3 Hangzhou Third People’s Hospital, Hangzhou 310009, China; 4 Key Laboratory of Neuropharmacology and Translational Medicine of Zhejiang Province, School of Pharmaceutical Sciences, Zhejiang Chinese Medical University, Hangzhou 310053, China

**Keywords:** shape effect, drug delivery, mesoporous silica nanoparticles (MSNs), anticancer therapy, biodistribution

## Abstract

A nanoparticle’s shape is a critical determinant of its biological interactions and therapeutic effectiveness. This study investigates the influence of shape on the performance of mesoporous silica nanoparticles (MSNs) in anticancer therapy. MSNs with spherical, rod-like, and hexagonal-plate-like shapes were synthesized, with particle sizes of around 240 nm, and their other surface properties were characterized. The drug loading capacities of the three shapes were controlled to be 47.46%, 49.41%, and 46.65%, respectively. The effects of shape on the release behaviors, cellular uptake mechanisms, and pharmacological behaviors of MSNs were systematically investigated. Through a series of in vitro studies using 4T1 cells and in vivo evaluations in 4T1 tumor-bearing mice, the release kinetics, cellular behaviors, pharmacological effects, circulation profiles, and therapeutic efficacy of MSNs were comprehensively assessed. Notably, hexagonal-plate-shaped MSNs loaded with PTX exhibited a prolonged circulation time (*t*_1/2_ = 13.59 ± 0.96 h), which was approximately 1.3 times that of spherical MSNs (*t*_1/2_ = 10.16 ± 0.38 h) and 1.5 times that of rod-shaped MSNs (*t*_1/2_ = 8.76 ± 1.37 h). This research underscores the significance of nanoparticles’ shapes in dictating their biological interactions and therapeutic outcomes, providing valuable insights for the rational design of targeted drug delivery systems in cancer therapy.

## 1. Introduction

In recent years, nanotechnology has emerged as a promising strategy to overcome technological bottlenecks across various scientific fields, with nano-medicine playing a pivotal role in the treatment of life-threatening diseases [1]. Compared to free drugs, they can be actively or passively targeted to disease sites, leading to more exciting therapeutic outcomes [2]. One major application involves loading therapeutic drugs onto nano-carriers and delivering them to target sites, offering a degree of controlled release and site specificity that free drugs cannot provide [3,4]. Taking cancer therapy as an example, nanoparticles can more easily penetrate cancerous regions through the abnormal vascular walls of tumor tissues, facilitating localized drug release. This can help maximize the drug concentration within tumor tissues while minimizing impacts on the surrounding normal tissues [5,6], and nano-carriers can effectively enhance the water solubility of drugs and slow down drug metabolism, thereby prolonging the circulation time of drugs in the body [7,8]. Furthermore, nanotechnologies such as molecularly imprinted polymers (MIPs) with diagnostic and targeted therapeutic capabilities; transdermal nanotechnology for skin cancer; lanthanide-based nanoparticles coated with specific peptides, ligands, and proteins targeting the tumor microenvironment; as well as magnetite nanoparticles for targeted drug delivery all show promising development [9,10,11,12]. These nanotechnologies provide drugs with a longer-lasting therapeutic effect, offering patients with breast cancer more sustainable treatment outcomes.

Nanoparticles exhibit strong appeal in drug delivery due to their typically superior physicochemical properties, which are attributed to their unique physical parameters such as size [13]. In the past two to three decades, researchers have primarily focused on adjusting the size and surface chemistry of nanoparticles to enhance their in vivo performance and elucidate their impacts on drug delivery [14,15]. For example, in studies on size, Sonavane et al. found that smaller nanoparticles exhibit longer circulation times and a higher accumulation in all organs, with smaller-sized nanoparticles even being capable of crossing the blood–brain barrier [16]. Despite active research in the field of nano-drug delivery systems, the extremely low clinical translation suggests that significant challenges remain in the study of nano-carriers [17]. Among these physical parameters, shape serves as a key physicochemical property of nano-carriers, largely determining their fate in vivo, and the impact of shape on drug delivery appears to be unclear. The shape of nano-carriers significantly influences various intracellular processes, such as cellular uptake, blood circulation, biodistribution, and disease targeting [18]. While most nano-carriers in preclinical or clinical studies are currently spherical, the unique properties of non-spherical nano-carriers may provide a new avenue for the rational design of nano-carriers for specific purposes. Research in this area holds profound significance for improving therapeutic outcomes, mitigating side effects, and designing applications of nanoscale delivery systems [19].

Mesoporous silica nanoparticles (MSNs) possess characteristics such as a high specific surface area, large pore volume, uniform porosity, stable aqueous dispersion, and biocompatibility, making them ideal carriers for biomedical applications [20]. Since the proposal of using the mesoporous material MCM-41 as a drug delivery system in 2001, silica-based materials, especially porous silica nanoparticles, have been considered promising drug delivery carriers [21]. The encapsulation of therapeutics in MSNs has been successfully utilized in the development of numerous new drug delivery vehicles [22]. Cancer is increasingly becoming a major threat to life worldwide, with breast cancer being the most common malignancy among women [23,24]. In breast cancer treatment, paclitaxel (PTX) serves as an efficient, low-toxicity, broad-spectrum natural anticancer drug, offering hope to patients. PTX works by disrupting the dynamics of tumor cell microtubules, thereby inhibiting cell growth and division by interfering with mitosis [25,26]. However, PTX’s hydrophobicity and systemic distribution limit its suitability as an effective anticancer drug [27]. Relevant studies have indicated that, compared to traditional PTX treatment, nanoparticle carriers can significantly increase the drug retention time in tumor tissues while reducing adverse reactions in patients [28,29]. Some paclitaxel-based nano-formulations, such as Abraxane and Lipusu, have begun to be applied in clinical therapy with favorable treatment outcomes. Compared to silica nanoparticles, nano-formulations like Abraxane and Lipusu may have better biocompatibility. However, these formulations still have inevitable side effects and may face more severe drug resistance issues, limiting their long-term therapeutic applications [30,31].

In this study, we utilized MSNs as templates to design nanoparticles of various shapes (spherical, rod-shaped, and hexagonal-plate-shaped). This choice was made due to the ease of controlling geometric shapes through synthesis, coupled with the inherent stability of nanoparticles. Paclitaxel (PTX) was used as a drug model for breast cancer treatment to compare drug release characteristics and therapeutic effects. The cellular uptake, uptake mechanisms, and organelle distribution differences of MSNs were evaluated in mouse breast cancer 4T1 cell lines using confocal laser scanning microscopy (CLSM) and flow cytometry (FCM), and the effects of PTX-loaded MSNs on proliferation and apoptosis were further investigated. In addition, the pharmacokinetics of MSNs loaded with PTX were evaluated in rats following intravenous injection compared to the use of a free PTX solution. A mouse breast cancer 4T1 cell line-derived xenograft model was established and used to assess anti-tumor efficacy.

## 2. Materials and Methods

### 2.1. Materials

Cetyltrimethylammonium bromide (CTAB), tetraethyl orthosilicate (TEOS), 3-aminopropyltriethoxysilane (APTES), and fluorescein isothiocyanate (FITC) were procured from Sigma-Aldrich (Saint Louis, MO, USA); lithocholic acid (LCA), N,N-dimethylformamide (DMF), chlorpromazine, amiloride, indocyanine green (ICG, 95%), ethynodiol diacetate, and silicon standard solution were sourced from Macklin (Shanghai, China); anhydrous ethanol, 25% ammonia solution, and paclitaxel standard (≥99%) were obtained from Aladdin (Shanghai, China); paclitaxel (PTX, 99%) was acquired from HEOWNS (Tianjin, China); RPMI-1640 medium, penicillin-streptomycin solution, phosphate-buffered saline (PBS), 0.25% trypsin (Trypsin-EDTA), and fetal bovine serum were purchased from Gibco (Saint Louis, MO, USA); the CCK-8 assay kit, 4′,6-diamidino-2-phenylindole (DAPI), and various organelle red probes were sourced from Beyotime (Shanghai, China); and methyl-β-cyclodextrin was obtained from Yuanye (Shanghai, China).

### 2.2. Preparation of MSNs with Different Shapes

MSNs with different shapes were fabricated using the template method. Briefly, for the preparation of a spherical MSN (MSN-S), 225 mg of CTAB was dissolved in 22.5 mL of ethanol, followed by the addition of 150 mL of deionized water. The pH was adjusted to 10.5 using a 1 M NaOH solution, and the mixture was homogenized and heated to 85 °C. Then, 1.25 mL of TEOS was added dropwise and stirred for 2 h [32]. The methods used for washing and removing template agents referenced those in previous studies in the literature [33]. After centrifugation at 18,000× *g* for 10 min, the white precipitate was collected and washed alternately with ethanol and water three times. After vacuum drying at 60 °C and grinding, the final MSN-S was obtained by high-temperature calcination at 550 °C for 6 h to remove CTAB.

For the rod-shaped MSN (MSN-R), we referenced previous methods and made modifications [34]. In brief, 450 mg of CTAB was dissolved in 100 mL of deionized water, followed by the addition of 2 mL of ammonia solution. After 1 h of stirring, we added 2.4 mL of TEOS dropwise and stirred it at room temperature for 20 h. The white precipitate was collected, and MSN-R was obtained through downstream procedures similar to the preparation of MSN-S.

For hexagonal-plate-shaped MSN (MSN-H), we referenced previous methods and made modifications [35]. In brief, a solution containing 50 mg of CTAB and 4.6 mg of LCA was dissolved in 25 mL of deionized water. Subsequently, 1.42 mL of ammonia solution was added, followed by uniform stirring. After the addition of 0.1 mL of ethanol and 15 min of stirring, 0.25 mL of TEOS was added dropwise, and the reaction was stirred for 90 min. The collected white precipitate was processed through downstream procedures similar to those for MSN-S to obtain MSN-H.

### 2.3. Characterization of MSNs with Different Shapes

#### 2.3.1. Particle Size and Zeta Potential

Particle size and zeta potential measurements were obtained by dynamic light scattering (DLS) using Zetasizer Nano (Malvern, UK). All measurements were performed at 25 °C after diluting the samples with deionized water to an appropriate volume.

#### 2.3.2. Morphology

The morphology of the samples was observed using an SU8010 scanning electron microscope (SEM) (HITACHI, Tokyo, Japan) and H-7650 transmission electron microscope (TEM) (HITACHI, Tokyo, Japan). For SEM, the powder of MSN was directly fixed onto the stub and sputter-coated with a thin layer of gold before observation. For TEM, the sample was initially diluted with deionized water to an appropriate volume. The resulting suspension was slowly dropped onto a 200-mesh carbon-coated copper grid, and the sample was observed after the drying process.

#### 2.3.3. Nitrogen Adsorption/Desorption Analysis and X-ray Diffraction

Nitrogen adsorption/desorption analysis was carried out on the ASAP2460 QuantaChrome instrument (Micromeritics, Norcross, GA, USA). All samples were dried at 300 °C for 8 h prior to analysis. The Barrett–Joyner–Halenda (BJH) method was used to obtain the specific surface area, pore volume, and pore size distribution from the adsorption branch of the isotherm [36].

For X-ray diffraction testing, an appropriate amount of dried mesoporous silica nanoparticles (MSN-S, MSN-R, and MSN-H) was taken for analysis. The testing conditions involved a 0.02-degree step scan ranging from 5 to 40 degrees at a rate of 2 degrees per minute to investigate the mesoporous structure.

#### 2.3.4. FTIR and TGA

Fourier-Transform Infrared Spectroscopy (FTIR) and Thermogravimetric Analysis (TGA) were performed using an Antaris II FTIR spectrophotometer (Thermo Fisher Scientific, Waltham, MA, USA) and TG 209 F3 thermogravimetric analyzer (NETZSCH, Free State of Bavaria, Germany) to confirm the complete removal of the template. For FTIR analysis, MSN samples were initially mixed with potassium bromide at a ratio of 1:30–1:100 and compressed into thin pellets for FTIR analysis. The wavelength scan range was set between 500 and 4000 cm^−1^. For TGA, tests were conducted under a nitrogen atmosphere, with a temperature range of 30~800 °C and a heating rate of 10 °C /min.

### 2.4. Control of Drug Loading

To prepare PTX-loaded MSN, an excess of PTX was added to ethanol to create a supersaturated solution. Subsequently, 10 mg of MSN with different shapes was dispersed in 1 mL of the oversaturated solution, and the dispersion was stirred at room temperature for 1 h. This process was followed by ultrasonic dispersion for 1 min. Drug loading of the three nanoparticle types was equalized by controlling the number of cycles. To remove unloaded PTX, particles were collected by centrifugation at 15,000× *g* for 15 min, washed three times with deionized water, and then dried at 60 °C [37].

To determine the drug loading efficiency of MSN, 10 mg of the loaded sample was dissolved in 5 mL of acetonitrile, and ultrasonic vibration was applied to facilitate the complete release of the drug from MSN. The solution was then centrifuged at 15,000 rpm for 15 min. The supernatant was determined using Waters H-class UPLC equipped with a UV detector by following a previously established program [38]. The detection wavelength was set at 227 nm, and the chromatographic column used was a CORTECS UPLC T3 Column (2.1 × 100 mm, 1.6 μm, Waters) maintained at a constant temperature of 30 °C. The mobile phase consisted of a mixture of acetonitrile and water in a volume ratio of 55/45 with a flow rate of 0.2 mL/min, and the injection volume was 2 μL. A linear relationship was established for PTX concentrations in the range of 1–200 μg/mL. The drug loading (DL) was calculated using the following formula:

DL = (amount of entrapped drug in MSN)/(weight of drug loaded MSN) × 100%

### 2.5. In Vitro Release of PTX

In vitro PTX release was tested by the dynamic dialysis method. An amount of 5 mg of PTX-loaded MSN with different shapes was dispersed in 2 mL of PBS at pH 7.4, and then sealed in dialysis bags (MWCO: 14 kDa). Subsequently, the dialysis bags were immersed in 30 mL of PBS at pH 7.4 and subjected to reciprocal shaking at 100 rpm while maintaining a constant temperature of 37 °C. At various time points, 1 mL of the supernatant was collected, and an equivalent volume of fresh PBS was replenished. The concentration of PTX was determined at 227 nm using Waters H-class UPLC equipped with a UV detector [33]. All tests were conducted in triplicate.

### 2.6. Cellular Recognition and Internalization

The intracellular localization of MSN was examined using confocal laser scanning microscopy (CLSM, FV 1200, Olympus, Tokyo, Japan). Briefly, 4T1 cells (1 × 10^5^ cells/well) were seeded in confocal culture dishes and cultured overnight. Then, the medium was replaced with culture medium containing FITC-labeled nanoparticles (FITC-MSN) at a concentration of 5 μM, followed by 4 h of incubation. Afterward, the cells were washed with PBS three times and stained with DAPI for 15 min. Finally, the fluorescence signals of each group were observed using CLSM [39].

Furthermore, 4T1 cells (2 × 10^5^ cells/well) were seeded in 6-well plates and cultured overnight. Then, the cells were treated with culture medium containing FITC at a concentration of 3 μmol/L or with culture medium containing FITC-labeled nanoparticles (FITC-MSN) for 4 h. Subsequently, the cells were washed with PBS three times and collected in flow tubes. Finally, the fluorescence intensity was detected using a CytoFlex flow cytometer (Beckman Coulter, Brea, CA, USA).

### 2.7. Endocytosis Inhibition Assays

The 4T1 cells (2 × 10^5^ cells/well) were seeded in 6-well plates and incubated overnight. Subsequently, they underwent the following treatments for 2 h: incubation at 4 °C to inhibit energy-dependent endocytosis; treatment with chlorpromazine (1 mg/mL) to inhibit clathrin-mediated endocytosis; treatment with methyl-β-cyclodextrin (0.5 mg/mL) to inhibit caveolae-mediated endocytosis; and treatment with amiloride (0.5 mg/mL) to inhibit macropinocytosis. Then, FITC-MSN was added to each group to achieve an FITC concentration of 10 μg/mL, followed by incubation in the dark for 4 h. The cells were then trypsinized, resuspended in 500 μL PBS, and analyzed using a CytoFlex flow cytometer [40].

### 2.8. Extracellular Organelle Distribution

The cellular organelle distribution of MSN was investigated using confocal laser scanning microscopy (CLSM, FV 1200, Olympus, Tokyo, Japan). In brief, 4T1 cells (1 × 10^5^ cells/well) were seeded in confocal culture dishes and cultured overnight. The medium was then replaced with culture medium containing FITC-MSN (FITC: 10 μg/mL), and the cells were further incubated for 4 h. Afterward, the cells were washed with PBS three times [41].

For lysosomes, cells were stained with Lyso-Tracker Red for 30 min; for microtubules, Tubulin-Tracker Red was used for staining for 45 min; for endoplasmic reticulum, ER-Tracker Red was employed for staining for 20 min; for the Golgi apparatus, Golgi-Tracker Red was used for staining for 30 min; and for mitochondria, Mito-Tracker Red CMXRos was applied for staining for 20 min. Finally, the fluorescent signals from each group were observed using CLSM.

### 2.9. Cytotoxicity

The cytotoxicity of a blank carrier MSN and a drug-loaded MSN on 4T1 cells was determined by a CCK-8 assay. 4T1 cells were cultured and tested in RPMI 1640 containing 10% FBS. The cells were seeded in a 96-well plate at a density of 1 × 10^4^ cells per well and allowed to incubate overnight (37 °C, 5% CO_2_, humidified). Subsequently, the cells were incubated with MSNs with different shapes, drug-loaded MSNs, and PTX solutions at various concentrations for 48 h, followed by addition of 10 μL CCK-8 reagent and incubation for 4 h at 37 °C. Cell viability was determined at 450 nm using a Synergy H1 microplate reader (BioTek, Winooski, VT, USA).

### 2.10. Cell Apoptosis Assay and Cycle Analysis

For cell apoptosis assay, 4T1 cells in logarithmic growth phase were seeded at a density of 5 × 10^5^ cells per well in a 6-well plate and incubated overnight at 37 °C. The cells were treated with MSNs with different shapes at an equivalent PTX concentration of 20 μg/mL for 48 h in triplicate with untreated cells serving as controls. After treatment, the cells were harvested by centrifugation and washed twice with cold PBS. Subsequently, the cells were resuspended in an equal volume of binding buffer, followed by staining with 5 μL of Annexin V-FITC and 10 μL of propidium iodide (PI) solution at room temperature for 15 min. The cell cycle was detected using a CytoFlex flow cytometer (Beckman Coulter, Brea, CA, USA).

For cell cycle analysis, 4T1 cells in the logarithmic growth phase were seeded at a density of 5 × 10^5^ cells per well in a 6-well plate and incubated overnight at 37 °C. The cells were treated with different shapes of MSN and PTX solution for 24 h in triplicate with untreated cells serving as a control. The cells were then precipitated, washed with ice-cold PBS, and fixed overnight at 4 °C in 70% (*v*/*v*) ethanol. Subsequently, the cells were centrifuged, washed with PBS, and stained with 500 μL propidium iodide (PI) solution at 37 °C for 30 min. The cell cycle was detected using a CytoFlex flow cytometer (Beckman Coulter, Brea, CA, USA) [42].

### 2.11. Three-Dimensional Tumor Spheroids Study

FITC-labeled nanoparticles were prepared as follows: In brief, 100 mg of MSN was dispersed in 50 mL of ethanol, and the mixture was heated to 45 °C. Then, 250 μL of water and 250 μL of APTES were sequentially added and stirred at 45 °C for 8 h. Amine-modified MSN (MSN-NH_2_) was synthesized through ultracentrifugation and purification. Subsequently, a mixture of 25 mg of MSN-NH_2_, 1.0 mg of FITC, and 5.0 mL of DMF was stirred in the dark at 25 °C for 12 h. FITC-labeled MSN (FITC-MSN) was collected by ultracentrifugation and washed with ethanol until the supernatant became colorless.

4T1 cells were incubated into ultra-low attachment plates. After 5 days, tumor cell spheres were treated with fresh culture medium containing FITC-MSN with different shapes (FITC: 10 μg/mL). Subsequently, the tumor spheres were incubated with FITC-MSN for 1–48 h. After washing with PBS, the samples were observed using confocal laser scanning microscopy (CLSM, Zeiss, Oberkochen, Germany) [43].

### 2.12. In Vivo Pharmacokinetic Study

To investigate the in vivo dynamics of PTX-loaded MSNs, the intravenous kinetics of the particles were studied in female SD rats weighing 200 ± 20 g. Twelve rats were randomly divided into 4 groups (*n* = 3) and administered with PTX-loaded MSN-S, MSN-R, MSN-H, and free PTX solution (10 mg PTX equivalent/kg, 1 mL) via tail vein injection. Blood samples (300 μL) were collected from the rats’ eye sockets at 5, 15, and 30 min and 1, 2, 4, 8, 12, 24, and 48 h post-injection into heparinized tubes. Subsequently, 100 μL of the blood sample was added to 600 μL of concentrated nitric acid, and after complete digestion, it was diluted to 7 mL with pure water. The silicon element content in the blood was quantified using ICP-MS (Thermo Fisher Scientific, Waltham, MA, USA) to quantify the nanoparticles in the blood [44]. 

For the quantification of PTX in the blood, 10 μL of 100 μg/mL norethindrone was added as an internal standard, and 200 μL of the blood sample was extracted with 2.5 mL of methanol. The mixture was vortexed for 2 min and then centrifuged at 4000 rpm for 10 min, and 2 mL of the supernatant was taken and dried by nitrogen. The residue was redissolved in 100 μL of acetonitrile. PTX concentration was determined by UPLC. Pharmacokinetic parameters were calculated using DAS 2.0 (China National Medical Products Administration, Beijing, China) [45].

### 2.13. In Vivo Imaging and Ex Vivo Distribution Analysis

The preparation of ICG-labeled nanoparticles is as follows: Following the procedure described in Section 2.8 for preparing MSN-NH_2_, 50 mg of MSN-NH_2_, 1 mg of ICG, and 10 mL of methanol were mixed thoroughly. The mixture was stirred in the dark at 25 °C for 12 h. ICG-labeled MSN (ICG-MSN) was collected by ultracentrifugation and eluted with ethanol until the supernatant was colorless.

Female BALB/c mice (4–6 weeks, 20 ± 2 g) were purchased from Zhejiang Chinese Medical University Laboratory Animal Research Center. To prepare 4T1 tumor-bearing mice, 4T1 cells in PBS buffer were subcutaneously injected to the left side of the back of each mouse at a dose of 1 × 10^6^ cells per mouse. Approximately 15 days later, once the tumor volume exceeded 150 mm3, the mice could be used for in vivo experiments. ICG or ICG-MSN with different shapes (ICG: 2 mg/kg) were injected into mice via the tail vein. At specified times (1, 2, 4, 8, 12, 24, and 48 h), ICG fluorescence was studied using an in vivo imaging system (excitation: 710 nm; filter: 745 nm; IVIS, PerkinElmer, Waltham, MA, USA). After 48 h post-injection, the mice were euthanized, and major organs (heart, liver, spleen, lungs, and kidneys) and tumors were harvested for ex vivo imaging.

### 2.14. In Vivo Anti-Tumor Evaluation

The anti-tumor efficacy of PTX-loaded MSN was tested using 4T1 tumor-bearing mice. The 4T1 tumor-bearing mice were randomly divided into five treatment groups (*n* = 6): saline control, free PTX, PTX-loaded MSN-S, PTX-loaded MSN-R, and PTX-loaded MSN-H (PTX: 10 mg/kg). The formulation was intravenously injected every three days for a total of 7 doses. Following the completion of dosing, observations continued for 7 days. During this period, body weights, tumor volumes, and survival rates were recorded every two days. Tumor volume was measured according to the following equation [46]:

$$\mathrm{Tumor}\;\mathrm{volume}=\frac{\mathrm{Tumor}\;\mathrm{length}\times {\mathrm{Tumor}\;\mathrm{width}}^{2}}{2}$$

Additionally, tumor tissues were collected for H&E, TUNEL, and Ki67 staining to assess tissue necrosis, apoptosis, and proliferation.

### 2.15. Biosafety Evaluation

After the treatment period, peripheral blood was collected from the orbital vein for blood routine and liver and kidney function analysis to evaluate the safety of the formulations. Subsequently, the mice were euthanized, and major organs and tumors were collected, fixed in 10% formaldehyde, embedded in paraffin, and sectioned for histopathological analysis [47].

### 2.16. Statistical Analysis

The data are presented as mean ± standard deviation (SD). Statistical significance of the means was assessed using one-way analysis of variance (ANOVA) with SPSS software (version 20.0, Chicago, IL, USA). In all studies, a *p*-value of less than 0.05 was considered statistically significant, and a *p*-value of less than 0.01 was considered highly significant.

## 3. Results and Discussion

### 3.1. Synthesis and Characterization of MSN with Different Shapes

Mesoporous silica nanoparticles (MSNs) with different shapes, namely MSN-S, MSN-R, and MSN-H, were synthesized using the template method. Three types of nanoparticles were prepared using cetyltrimethylammonium bromide (CTAB) as the soft template and tetraethyl orthosilicate (TEOS) as the silicon source; however, they differed in the choice of alkaline catalyst. MSN-S used NaOH as the basic catalyst, while MSN-R and MSN-H used ammonia solution. Additionally, MSN-H employed lithocholic acid (LCA) as a structure-directing agent.

**Figure 1:**
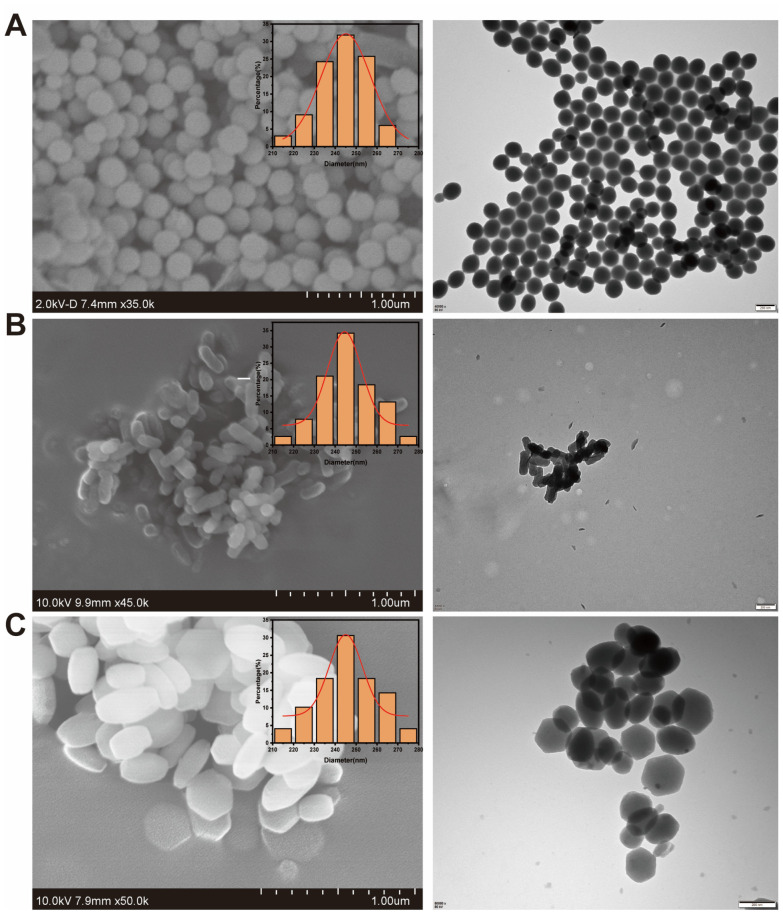
Morphology of MSN with different shapes. Scanning Electron Microscopy (SEM) images and size distribution, as well as Transmission Electron Microscopy (TEM) images of (**A**) MSN-S, (**B**) MSN-R, and (**C**) MSN-H.

The SEM and TEM images (Figure 1) revealed that MSN-S exhibited a spherical shape with a diameter of approximately 230 nm, MSN-R displayed a rod-like morphology with a long diameter of around 240 nm and a short diameter of about 80 nm, while MSN-H appeared as a hexagonal platelet with a diameter of about 210 nm and a thickness of approximately 30 nm. Dynamic light scattering measurements showed that the particle sizes of MSN-S, MSN-R, and MSN-H were all around 240 nm, with polydispersity indices (PDIs) of 0.225 ± 0.012, 0.260 ± 0.016, and 0.251 ± 0.008. The zeta potentials were approximately −25.4 ± 1.6, −22.8 ± 0.8, and −21.9 ± 1.3 mV, respectively, and all around −23 mV. The conductivity of the MSN-S solution was approximately 4.25 × 10^−3^ mS/cm, the conductivity of the MSN-R solution was approximately 8.22 × 10^−3^ mS/cm, and the conductivity of the MSN-H solution was approximately 5.48 × 10^−3^ mS/cm. Although smaller particles (≤100 nm) are more favorable for crossing various biological barriers, the nanoparticle sizes were fixed at around 240 nm for comparison due to the difficulty in manufacturing non-spherical nanoparticles [48].

**Figure 2:**
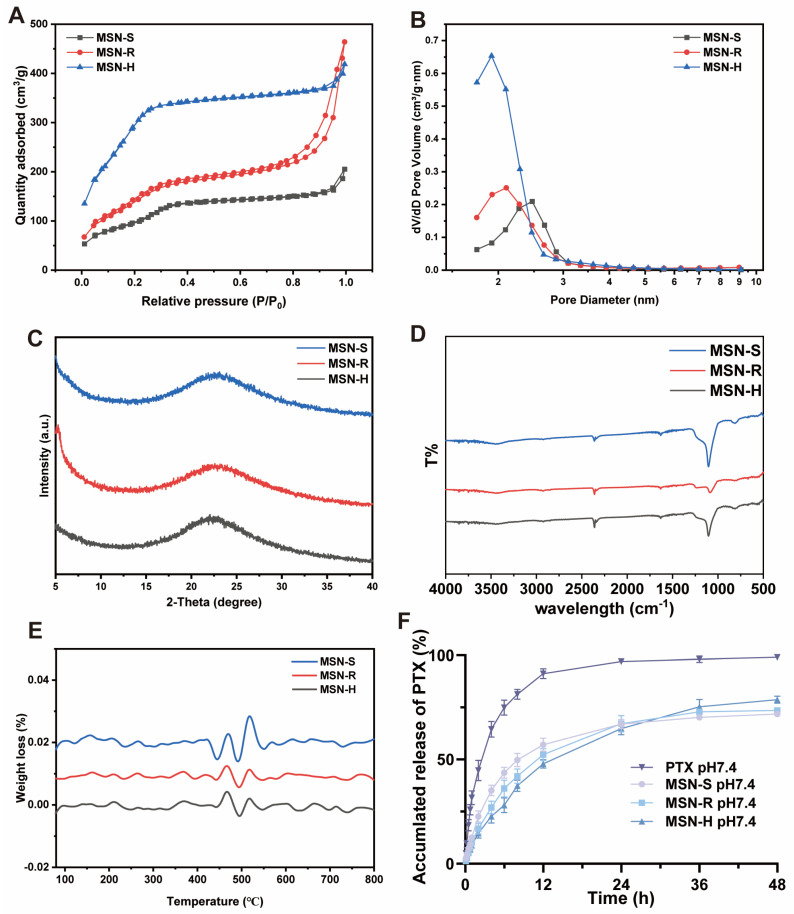
Characterization of MSN with different shapes. (**A**) Nitrogen adsorption–desorption isotherms. (**B**) Pore size distribution curves. (**C**) Wide-angle X-ray diffraction (XRD) patterns. (**D**) Infrared spectra. (**E**) Thermogravimetric analysis curves. (**F**) In vitro release profiles of paclitaxel (PTX) from MSN loaded with PTX of different shapes in PBS at 37 °C (*n* = 3).

The nitrogen adsorption–desorption isotherms for all three types of MSNs exhibited type IV isotherms (Figure 2A), indicating the presence of mesoporous structures in all tested MSNs [49]. The pore size distribution results (Figure 2B) show that the average pore sizes of the three MSNs were similar, ranging from 2 to 3 nm. Wide-angle X-ray diffraction (Figure 2C) revealed distinct characteristic peaks at around 23° for MSN-S, MSN-R, and MSN-H, corresponding to the characteristic peaks of mesoporous silica. These results confirm that MSN-S, MSN-R, and MSN-H possess ordered mesoporous silica structures [50].

In the FTIR spectra (Figure 2D), all three varieties of nanoparticles display consistent absorption peaks. Notably, prominent peaks were observed for Si-O-Si bonding at 1109 cm^−1^, for Si-OH at 813 cm^−1^, and for OH at 1613 cm^−1^, with the absence of characteristic signals from CTAB and LCA. The TGA results (Figure 2E) showed almost no change in weight for the tested MSNs between 80 and 800 °C. Both the FTIR and TGA results indicate the complete removal of the templates [51].

### 3.2. Encapsulation of PTX and In Vitro Drug Release from MSN

The effective encapsulation of PTX within MSNs may be attributed to the high surface-area-to-volume ratio of MSNs and the electrostatic interactions between PTX and MSNs [52]. Considering potential variances in the drug-loading capacity among MSNs of varying shapes, an excess of paclitaxel (PTX) was initially dissolved in acetonitrile to achieve a state of supersaturation with PTX. Subsequently, 10 mg of differently shaped MSN was added, and the mixture underwent an ultrasound treatment for 1 min, followed by stirring for 1 h per cycle, aiming to regulate the drug loading based on the number of cycles. In our preliminary investigation, it was observed that with eight cycles for MSN-S, six cycles for MSN-R, and five cycles for MSN-H, the drug loading remained consistent at approximately 47.46%, 49.41%, and 46.65%, respectively.

The in vitro release of PTX from MSNs was compared in pH 7.4 PBS at 37 °C (Figure 2F). PTX was gradually released from MSNs according to first-order kinetics, with cumulative release percentages of 91.17 ± 2.34% for PTX, 57.06 ± 3.07% for MSN-S, 52.30 ± 2.87% for MSN-R, and 47.88 ± 2.02% for MSN-H within 12 h. The findings suggest that drug loading in MSNs can delay drug release, and there are differences in the release rates among MSNs with different shapes, with the sequence of release rates being MSN-S > MSN-R > MSN-H. Such variance could be attributed to the potentially enhanced stability resulting from the steric hindrance in plate-shaped nanoparticles compared to their spherical and rod-shaped counterparts. This increased stability may contribute to a slower dissociation of PTX from the silica matrix, resulting in a slower release profile [53].

### 3.3. The Shapes of MSNs Influence Their Cellular Uptake, Uptake Mechanisms, and Subcellular Organelle Distributions

**Figure 3:**
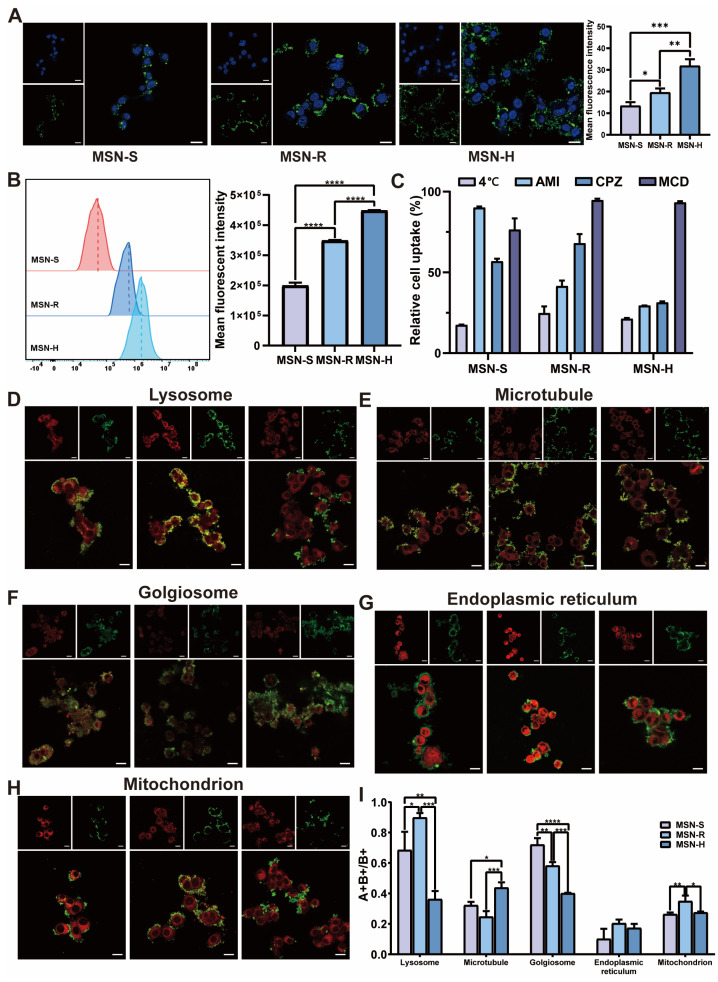
Cellular behavior studies. (**A**) Confocal laser scanning microscopy (CLSM) images and fluorescence analysis (*n* = 3) of internalization of MSN with different shapes by 4T1 cells. (**B**) Quantitative analysis by flow cytometry (FCM). (**C**) Investigation of uptake mechanisms of differently shaped MSN by 4T1 cells. Results of (**D**) lysosomal distribution, (**E**) microtubules, (**F**) Golgi apparatus, (**G**) endoplasmic reticulum, (**H**) mitochondria, and (**I**) fluorescence quantification for MSN-S, MSN-R, and MSN-H. Scale bar: 20 μm (*: *p* < 0.05, **: *p* < 0.01, ***: *p* < 0.005, ****: *p* < 0.001).

The cellular uptake of three types of nanoparticles labeled with FITC was investigated using both the CLSM and FCM methods. The results from the CLSM and Image J fluorescence analysis (Figure 3A) demonstrate discernible discrepancies in the average fluorescence signals among the three nanoparticle types, with the fluorescence intensity ranking being MSN-H > MSN-R > MSN-S. A further quantitative comparison of the cellular uptake of the three types of nanoparticles was performed using FCM (Figure 3B). The results reveal statistically significant differences (*p* < 0.0001) in the cellular uptake of nanoparticles by 4T1 cells at 37 °C, with the uptake sequence being MSN-H > MSN-R > MSN-S, which is consistent with the CLSM observations [54]. Therefore, the nanoparticle shape affects cellular uptake, with hexagonal-plate-shaped nanoparticles being more readily taken up by 4T1 cells, followed by rod-like and spherical nanoparticles.

After treatment at 4 °C and the addition of endocytosis inhibitors (amiloride, chlorpromazine, and methyl-β-cyclodextrin) with PBS as the control, the cellular uptake mechanisms of nanoparticles with different shapes by 4T1 cells were explored using FCM. The results in Figure 3C indicate that after treatment at 4 °C, the uptake of all three types of nanoparticles was significantly affected. Therefore, the uptake of the three types of nanoparticles by 4T1 cells occurred through an energy-dependent pathway. However, there were differences in the uptake mechanisms of nanoparticles of different shapes. Specifically, 4T1 cells primarily utilized clathrin-mediated endocytosis with caveolin-mediated endocytosis as a secondary pathway for the uptake of MSN-S. For MSN-R, macropinocytosis was the primary mechanism of uptake, with a concomitant involvement of clathrin-mediated endocytosis. MSN-H was taken up through both macropinocytosis and clathrin-mediated endocytosis. Therefore, differently shaped nanoparticles exhibit distinct uptake mechanisms, leading to differences in cellular uptake [55].

Through the utilization of five different red fluorescence probes targeting mitochondria, lysosomes, endoplasmic reticulum, microtubules, and the Golgi apparatus, the cellular uptake and distribution variances of nanoparticles of different shapes were quantitatively analyzed using CLSM and Image J (Version 1.53a, National Institutes of Health, Bethesda, MD, USA) (Figure 3D–I). The results indicate that nanoparticles are less likely to enter the endoplasmic reticulum, while their distribution within lysosomes, microtubules, the Golgi apparatus, and mitochondria differs significantly. Lysosomes play a crucial role in engulfing foreign particles and subsequently degrading them, underscoring the significance of localization for nanomaterials [56]. Figure 3D,I demonstrate a higher distribution of nanoparticles within lysosomes, with MSN-R being the most readily engulfed, followed by MSN-S, while MSN-H is the least prone to lysosomal engulfment. Given that the principal site of action for paclitaxel loaded in this investigation is microtubules, the nanoparticles affixed to microtubules discharge the drug, thereby promoting its pharmacological efficacy [57]. Figure 3E,I reveal that hexagonal-plate-shaped nanoparticles are more prone to adhere to microtubules, while there is no significant difference in the ability of rod-shaped and spherical nanoparticles to bind to microtubules. An association exists between the Golgi apparatus and exocytosis, as nanoparticles have the potential to be extruded from cells into the extracellular milieu via the exocytic process [58]. Figure 3F,I indicate that MSN-S is the most likely to be captured by the Golgi apparatus, followed by MSN-R, while MSN-H is relatively less likely to be captured by the Golgi apparatus. Furthermore, compared to MSN-S and MSN-H, MSN-R is more likely to enter mitochondria, while there is no statistical difference in the fluorescence quantification values of MSN-S and MSN-H within mitochondria [59]. In summary, nanoparticles with different shapes exhibit variations in their distribution within cellular organelles. Therefore, when designing nano-carriers, considering the nanoparticle shape based on the sites of action for different drugs can optimize therapeutic efficacy.

### 3.4. The Shapes of PTX-Loaded MSNs Affects Their Ability to Inhibit Cell Proliferation, Promote Apoptosis, and Arrest the Cell Cycle

**Figure 4:**
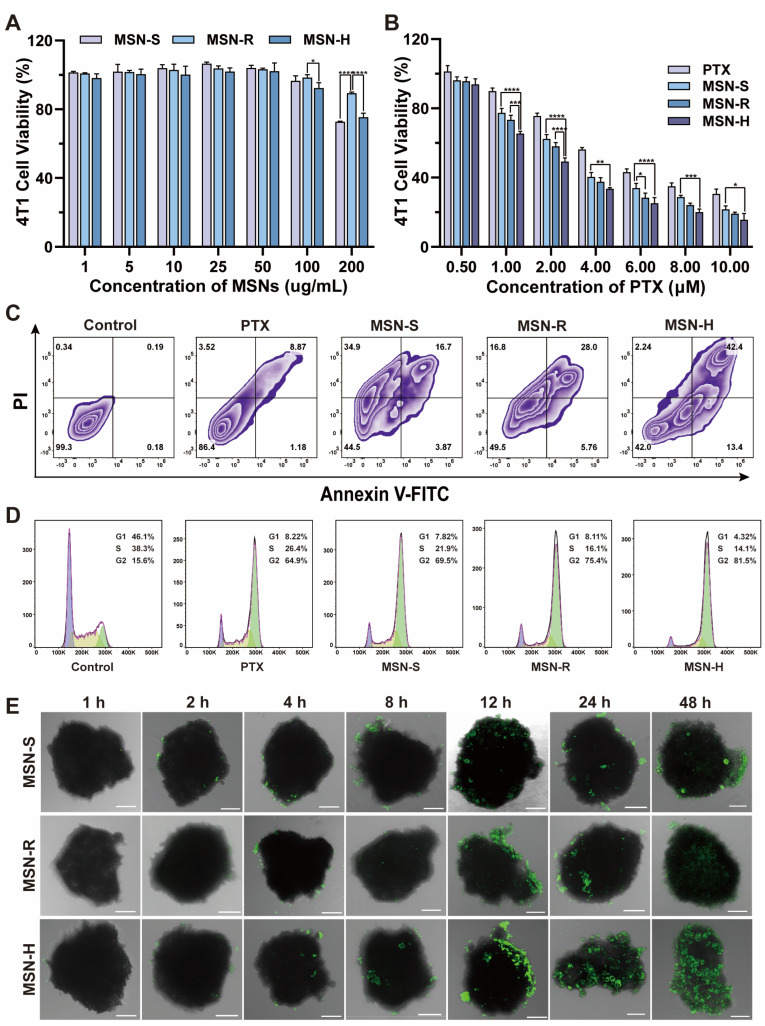
Cellular pharmacology studies. (**A**) Cell viability of 4T1 cells incubated with (**A**) empty carriers of different shapes at different concentrations and (**B**) PTX-loaded MSNs after 48 h (*n* = 3). (**C**) Apoptosis of 4T1 cells after 48 h of incubation with PTX-loaded MSNs. (**D**) Cell cycle analysis of 4T1 cells after 24 h of incubation with PTX-loaded MSNs of different shapes, purple for G1 phase, yellow for S phase, and green for G2 phase. (**E**) Confocal laser scanning microscopy (CLSM) images of three-dimensional tumor spheroids incubated with MSNs of different shapes for 48 h, scale 100 μm. (*: *p* < 0.05, **: *p* < 0.01, ***: *p* < 0.005, ****: *p* < 0.001).

A Cell Counting Kit-8 (CCK-8) assay was used to assess the influence of both blank and drug-loaded MSNs on the proliferation of 4T1 cells. For the blank carriers, as the concentration increased, the relative cell viability decreased (Figure 4A). All MSN groups displayed a degree of cytotoxicity, with MSN-H demonstrating the most pronounced cytotoxic effect, followed by MSN-S, while MSN-R exhibited a comparatively minimal variation. This may be due to the fact that MSN-R is more easily engulfed by lysosomes and subsequently degraded and digested, resulting in relatively weaker cytotoxicity. For the drug-loaded nanoparticles (Figure 4B), the IC50 values of the PTX-loaded MSN-S, MSN-R, MSN-H, and free PTX solution against 4T1 cells were 3.234 ± 0.210, 2.755 ± 0.016, 2.181 ± 0.048, and 4.996 ± 0.101 μM, respectively. Compared to the free PTX solution, MSNs of different shapes exhibited higher anti-proliferative efficiency at different drug concentrations with lower IC50 values. This observation could be attributed to the increased cellular uptake of MSNs compared to free drugs, resulting in the heightened cytotoxicity of PTX within tumor cells [60]. Moreover, MSN-H exhibited the lowest IC50 value against 4T1 cells, with significant differences compared to other groups, indicating that drug-loaded MSN-H has the strongest inhibitory effect on tumor cells.

Cell apoptosis experiments were conducted using flow cytometry to investigate cell death after treatment with drug-loaded MSNs and a PTX solution (Figure 4C). At the same PTX concentration, the proportion of viable cells in each MSN group was significantly lower than that in the PTX solution group, indicating a higher internalization of MSNs by 4T1 cells, thereby enhancing their anti-proliferative effect. Furthermore, the percentages of early and late apoptotic cells in the PTX-loaded MSN-H group were notably higher compared to those of the MSN-S and MSN-R groups. This observation could be attributed to variances in the cellular uptake levels and intracellular distribution.

Cell cycle distribution was assessed using propidium iodide (PI) staining and flow cytometry, with 4T1 cells being treated with drug-loaded MSNs of different shapes and a PTX solution at the IC50 concentration (Figure 4D). As a type of taxane alkaloid anticancer drug, PTX can stabilize microtubules by disrupting the dynamic equilibrium of tubulin protein and tubulin protein dimers, resulting in cell arrest at the G2 phase, thereby inhibiting cancer cell mitosis and triggering apoptosis [61,62]. At the same PTX concentration, the proportion of G2 phase cells in the PTX solution group was the lowest at 64.9% compared to each MSN group. Additionally, the proportion of G2 phase cells in the MSN-H group was higher than those in the MSN-S and MSN-R groups.

In summary, shape influences the cellular uptake mechanism of nanoparticles, with MSN-H entering cells in greater quantities, being less engulfed and digested by lysosomes after entering cells and accumulating more at microtubules. Therefore, it is easier to release PTX at microtubules inside cells, resulting in a stronger therapeutic effect.

### 3.5. The Shapes of MSNs Influence Their Penetration Capability in 3D Tumor Spheroids

Three-dimensional tumor spheroids were employed to investigate the penetration of MSNs with different shapes [63]. As shown in Figure 4E, for MSN-H, fluorescent signals were observed inside the tumor spheroids after 8 h of incubation, with a significant increase in signal intensity at 12 h. However, the signal at the central position remained relatively weak. By 24 h, MSN-H had completely penetrated into the tumor spheroids. For MSN-R, no obvious fluorescent signals were detected inside the tumor spheroids within the first 12 h of incubation, but signals appeared internally by 24 h, albeit with a weak central signal. By 48 h, MSN-R had fully penetrated the tumor spheroids, although with a lower overall intensity. In the case of MSN-S, there were no apparent fluorescent signals within the tumor spheroids during the initial 12 h of incubation. However, noticeable fluorescent signals emerged internally at 12 h, and by 24 h, MSN-S had completely penetrated into the tumor spheroids. Although the penetration increased by 48 h, the overall signal intensity remained weak. Therefore, the shapes of nanoparticles indeed affected their penetration into tumor spheroids. MSN-H demonstrated faster and more extensive penetration compared to MSN-S and MSN-R. While the penetration rates were similar between MSN-S and MSN-R, MSN-S exhibited higher penetration levels than MSN-R.

### 3.6. The Shapes of PTX-Loaded MSNs Affect Their In Vivo Circulation and Drug Release

To maintain long-term therapeutic effects, drug delivery systems need to circulate in the bloodstream for an extended period [64]. Therefore, we investigated the in vivo pharmacokinetics of a free PTX solution and PTX-loaded MSNs of different shapes in SD rats after intravenous injection. The plasma concentration of PTX was determined by UPLC, while the concentration of MSNs in the blood samples was represented by the silicon content measured using ICP-MS (with blank plasma as the baseline).

**Figure 5:**
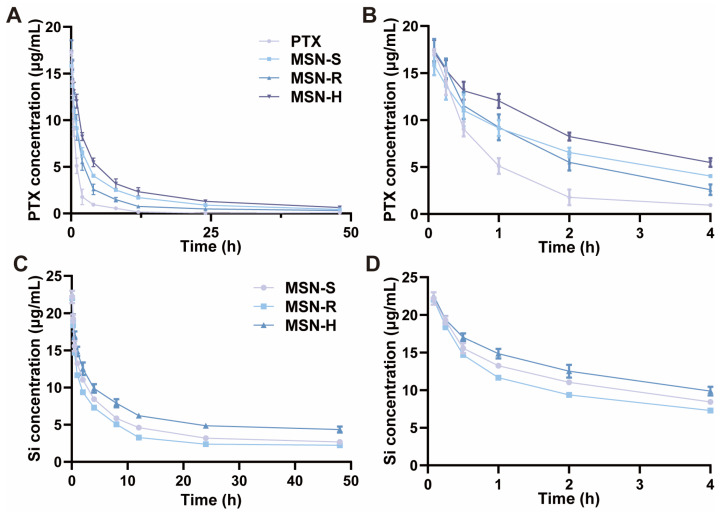
In vivo pharmacokinetic curves. SD rats (*n* = 3) were intravenously injected with MSN-S, MSN-R, MSN-H, and PTX solution at equivalent dose of 5 mg/kg of PTX. (**A**) Pharmacokinetic curves of PTX in blood. (**C**) Pharmacokinetic curves of MSN in blood. (**B**,**D**) Enlarged views corresponding to 0–4 h portion in (**A**) and (**C**), respectively.

The pharmacokinetic curves of PTX showed that within 48 h after a single injection, PTX-loaded MSNs of different shapes exhibited a slower decline in the bloodstream compared to the PTX solution, which exhibited a notably short half-life, indicating rapid clearance from the bloodstream (Figure 5A,B and Table 1). Considering the in vitro drug release characteristics, the prolonged circulation in the bloodstream might be attributed to the delayed release of PTX from the mesoporous matrix of MSN [65]. As an indicator of blood retention [66], the AUC of PTX for MSN-H (111.41 ± 6.68 mg/L·h) was approximately five times greater than that of the PTX solution (23.75 ± 3.95 mg/L·h). Furthermore, MSN-H exhibited the longest retention time, which was significantly higher than that of MSN-S (83.33 ± 5.51 mg/L·h) and MSN-R (57.77 ± 4.75 mg/L·h).

An analysis of MSNs based on ICP-MS showed that all MSNs could be retained in the bloodstream for over 48 h after a single injection (Figure 5C,D and Table 2). The AUC of MSN-H was 289.24 ± 7.41 mg/L·h, which was higher than that of MSN-R (173.87 ± 2.83 mg/L·h) and MSN-S (213.73 ± 3.72 mg/L·h), indicating that the order of efficacy was MSN-H > MSN-S > MSN-R. Therefore, hexagonal-plate-shaped nanoparticles exhibited longer circulation times in the bloodstream. On one hand, spherical nanoparticles are readily phagocytosed by macrophages. In contrast, the internalization of non-spherical nanoparticles necessitates adsorption along their long and short axes. Plate-shaped nanoparticles exhibit a larger local curvature than rod-shaped ones, which demands higher energy consumption for phagocytosis by macrophages, rendering it more challenging for macrophages to engulf plate-shaped nanoparticles [67]. On the other hand, the lateral displacement of non-spherical nanoparticles within blood vessels is influenced by the direction of particle orientation during flow. Plate-shaped particles can traverse highly oscillating trajectories within blood vessels, displaying enhanced adherence to the vessel wall compared to rod-shaped and spherical nanoparticles [68,69]. Consequently, this adherence prolongs the circulation time in vivo. Therefore, the shape of nanoparticles indeed exerts a discernible influence on their circulation within the bloodstream, with MSN-H notably enhancing nanoparticle blood circulation.

### 3.7. The Shapes of MSNs Influence Their Biological Distribution and Tumor-Targeting Efficiency

**Figure 6:**
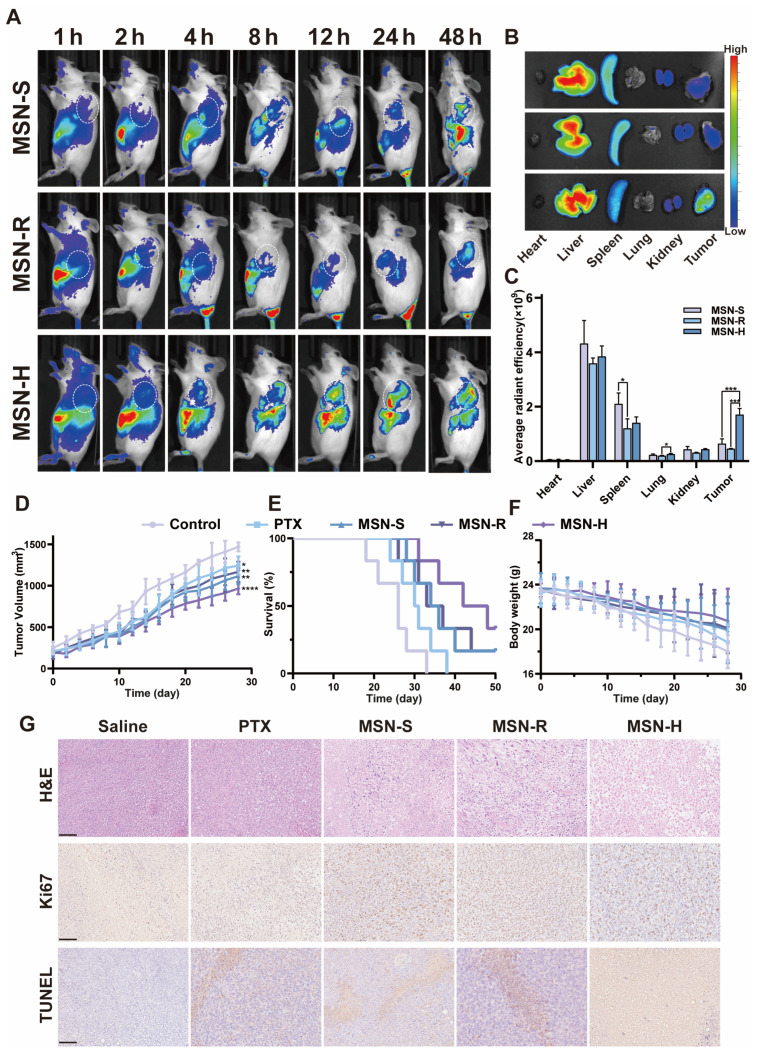
In vivo distribution and pharmacodynamics study. (**A**) In vivo fluorescence distribution images after injection of MSN-S, MSN-R, and MSN-H in tumor-bearing mice, (**B**) tissue distribution at 48 h, and (**C**) semi-quantitative fluorescence analysis. (**D**) Relative tumor volumes, (**E**) survival rates, and (**F**) body weights of mice treated with saline, PTX solution, PTX-loaded MSN-S, and MSN-R; (**G**) H&E, Ki67, and TUNEL staining of tumor tissues. Scale bar: 100 μm. * *p* < 0.05; ** *p* < 0.01, ***: *p* < 0.005, ****: *p* < 0.001.

Subsequently, we utilized ICG-MSN to investigate the real-time and ex vivo tumor and organ distribution of MSNs with different shapes [70]. MSNs swiftly dispersed throughout the body, with prominent accumulation initially observed in the liver, gradually transitioning to accumulation at the tumor site over time (Figure 6A). MSN-S began to aggregate in the liver at 2 h, slowly accumulating at the tumor site by 4 h, and started to be excreted from the body at 8 h, with significantly higher accumulation in the liver and in areas other than the tumor site at 24 h. MSN-R showed substantial accumulation in the liver at 1 h, followed by slow accumulation at the tumor site and subsequent metabolism or excretion from the body at 24 h. MSN-H exhibited systemic distribution at 1 h, began to accumulate at the tumor site at 2 h, showed noticeable fluorescence signals at the tumor site by 4 h, and predominantly accumulated in the liver and tumor sites by 12 h, with continued concentration in the liver and tumor sites at 48 h.

Fluorescence imaging and analysis of ex vivo tissues from mice at 48 h (Figure 6B,C) revealed that MSNs from all groups primarily accumulated in the liver, tumor, and spleen. The tumor fluorescence intensity of MSN-H was significantly higher than that of MSN-S and MSN-R. The fluorescence intensity in the liver was relatively the weakest for MSN-R, suggesting that MSN-R tends to accumulate in the liver and be excreted from the body. MSN-S exhibited the strongest fluorescence intensity in the spleen, which may be attributed to the propensity of spherical nanoparticles larger than 200 nm to be engulfed by the mononuclear phagocyte system (MPS) of the liver and spleen, with non-spherical nanoparticles demonstrating a greater ability to evade capture by the MPS [71,72].

### 3.8. The Shapes of PTX-Loaded MSNs Affect Their In Vivo Anticancer Efficacy

To further evaluate the anti-tumor efficacy of PTX-loaded MSNs, an in vivo study was conducted using 4T1 tumor-bearing mice [73]. Over a period of 28 days, the tumor volumes in all groups showed a trend of growth. As depicted in Figure 6D, the tumor volumes in each treatment group were smaller than those in the control group, with statistically significant differences observed (*p* < 0.05). Compared to the free PTX group, all MSN-treated groups exhibited superior tumor suppression effects, with MSN-H demonstrating the most potent inhibition, while the differences in the tumor volumes between MSN-R and MSN-S were relatively minor. Additionally, the survival curve results within 50 days (Figure 6E) show that all mice in the control group died by day 33. In contrast, treatment with MSN-H significantly prolonged the survival period of tumor-bearing mice and increased their survival rates compared to other treatment groups. A preliminary assessment of systemic toxicity using body weight as an indicator (Figure 6F) revealed a degree of weight loss in all groups, with the slowest decline being observed in the MSN-H group, although no significant differences were observed compared to other groups.

Furthermore, the in vivo anti-tumor effects of different treatment groups were further validated through a pathological examination (Figure 6G). The H&E staining results show more severe vacuolization in all MSN-treated groups compared to the PTX group, with variations being observed among the different MSN groups, and with MSN-H exhibiting the most severe vacuolization. The Ki67 and TUNEL staining results indicate that, compared to the control group, all treatment groups exerted certain inhibitory effects on tumor tissue proliferation and promoted apoptosis. Moreover, the therapeutic effects of all MSN-treated groups were superior to those of the free drug group, with MSN-H demonstrating the best anti-tumor efficacy.

### 3.9. The Influence of Shape on the In Vivo Safety of PTX-Loaded MSNs

**Figure 7:**
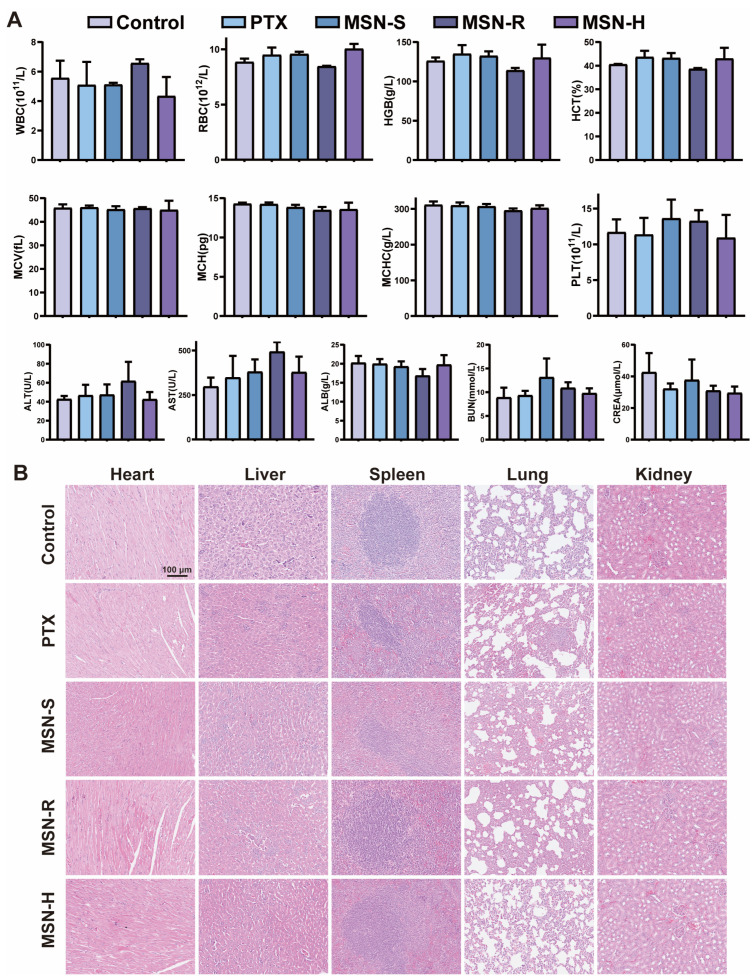
In vivo safety study. (**A**) Hematological and liver–kidney function indicators of mice after different treatment regimens, and (**B**) H&E staining of major tissues.

The safety of the mice in each group was assessed through hematological and serum biochemical analyses [47] (Figure 7A). There were no significant abnormalities observed in the blood routine and liver and kidney function indicators of mice in each group. Although some indicators, such as alanine aminotransferase (ALT) and aspartate aminotransferase (AST), were slightly higher in the MSN-R group compared to the other groups, these differences were not clinically significant. We speculate that this may be due to the rapid accumulation of MSN-R in the liver and its primarily hepatic metabolism, leading to slight liver damage in mice. It is worth noting that no significant signs of damage were observed in the major organ tissues upon examination using H&E staining (Figure 7B). This result further confirms the excellent biocompatibility and safety of each treatment group in vivo.

These findings suggest that while PTX-loaded MSNs exhibit excellent efficacy in anti-tumor therapy, their biological impact on the organism is relatively minor. Therefore, these MSNs hold promise as effective and safe drug carriers for the future treatment of tumors and other diseases.

## 4. Conclusions

In our investigation, we meticulously synthesized three distinct morphologies of mesoporous silica nanoparticles (MSNs), including spherical, rod-shaped, and hexagonal-plate-shaped structures. Rigorous physical characterization procedures corroborated the uniformity of particle size, morphology, and surface features across the MSN variants. Through a comprehensive series of both in vitro and in vivo assessments, encompassing analyses conducted on 4T1 cell cultures and 4T1 tumor-bearing mouse models, we uncovered the profound impact of nanoparticle shapes on diverse aspects of their performance. Our findings illuminated how the specific shapes of the nanoparticles significantly influenced critical parameters, such as the rate and pattern of drug release in vitro, cellular interactions and behaviors, pharmacological responses within cells, as well as the circulation kinetics and therapeutic efficacy in vivo. Notably, hexagonal-plate-shaped MSNs emerged as particularly promising, exhibiting prolonged circulation times and delivering superior therapeutic outcomes compared to their spherical and rod-shaped counterparts. Overall, our study underscores the pivotal role of nanoparticle morphology in dictating the effectiveness of drug delivery systems and tumor-targeted therapies. These insights hold considerable promise for informing the rational design and optimization of future targeted drug delivery platforms.

## Figures and Tables

**Table 1:** Pharmacokinetic parameters of PTX (mean ± SD, *n* = 3).

Formulation	AUC_0−t_ (mg/L × h)	MRT_0−∞_ (h)	*t*_1/2_ (h)
PTX	23.75 ± 3.95	6.03 ± 2.59	5.35 ± 1.85
MSN-S	83.33 ± 5.51	15.79 ± 1.32	10.16 ± 0.38
MSN-R	57.77 ± 4.75	13.90 ± 2.57	8.76 ± 1.37
MSN-H	111.41 ± 6.68	18.48 ± 0.67	13.59 ± 0.96

**Table 2:** Pharmacokinetic parameters of MSNs (mean ± SD, *n* = 3).

Formulation	AUC_0−t_ (mg/L × h)	MRT_0−∞_ (h)	*t*_1/2_ (h)
MSN-S	213.73 ± 3.72	33.37 ± 1.46	15.60 ± 1.03
MSN-R	173.87 ± 2.83	30.19 ± 1.27	12.99 ± 0.42
MSN-H	289.24 ± 7.41	41.60 ± 2.77	18.90 ± 0.43

## Data Availability

The original contributions presented in the study are included in the article, further inquiries can be directed to the corresponding authors.
